# Features of serum fatty acids in acute ischaemic stroke patients aged 50 years or older

**DOI:** 10.1186/s12872-020-01408-1

**Published:** 2020-03-10

**Authors:** Takahisa Mori, Kazuhiro Yoshioka

**Affiliations:** Department of Stroke Treatment, Shonan Kamakura General Hospital Stroke Centre, Okamoto 1370-1, Kamakura City, Kanagawa 247-8533 Japan

**Keywords:** Serum fatty acids, Serum lipids, Acute ischaemic stroke, Elderly patients

## Abstract

**Background:**

Serum fatty acid (s-FA) compositions and their correlation with serum lipids (s-LPs) such as total cholesterol (T-CHO) and triglycerides (TG) have been reported in healthy young subjects. However, little is known about such features in acute ischaemic stroke (AIS). The aim of our study was to investigate s-FA characteristics and their correlation with AIS in elderly patients.

**Methods:**

We conducted a cross-sectional study of patients aged 50 years or older who were admitted between September 2015 and March 2017 within 24 h of the first AIS onset. We evaluated concentrations and compositions of s-FAs and their association with s-LPs, age, and ischaemic stroke subtypes, including large-artery atherosclerosis (LAA), small-vessel occlusion (SVO), and cardioembolism (CE) or others.

**Results:**

One hundred ninety-one patients met our inclusion criteria. Their average age was 74.4 years, mean T-CHO and median TG were 203.4 and 94.5 mg/dl, respectively, and median or mean concentrations of palmitic acid (PA), oleic acid (OlA), linoleic acid (LiA), and docosahexaenoic acid (DHA) were 680.7, 602.5, 795.2, and 136.9 μg/ml, respectively, with mean compositions of 23.7, 21.3, 27.1, and 4.4%, respectively. PA, OlA, and LiA concentrations were weakly negatively associated with age and positively correlated with TG. In LAA or SVO (LAA_SVO) and CE or others (CE_O), mean age was 71.9 and 77.4 years (*p* < 0.001), mean T-CHO was 213.9 and 191.2 mg/dl (*p* < 0.0001), median TG was 106.5 and 88.5 mg/dl (*p* < 0.01), median PA was 717.2 and 648.4 μg/ml (*p* < 0.01), median OlA was 638.2 and 567.5 μg/ml (*p* < 0.01), and median LiA was 844.7 and 728.5 μg/ml (*p* < 0.01), respectively. DHA composition was weakly positively correlated with age. There were no differences in PA, OlA, LiA, and DHA compositions between LAA_SVO and CE_O.

**Conclusions:**

In AIS elderly patients, concentrations, rather than compositions of PA, OlA, and LiA, correlated with age, TG, and ischaemic stroke subtypes. Patients with LAA_SVO were younger and had higher concentrations of PA, OlA, and LiA than those with CE_O. There were no differences in such compositions between LAA_SVO and CE_O.

## Background

Previous studies have reported compositions of serum fatty acids (s-FAs) such as saturated fatty acids (SFAs), n-6 polyunsaturated fatty acids (n-6 PUFAs), n-9 monounsaturated fatty acid (n-9 MUFA), and n-3 polyunsaturated fatty acids (n-3 PUFAs), and their correlation with serum lipids (s-LPs) in healthy young or middle-aged human subjects [1–5]. The proportion of n-6 PUFAs, as a fraction of total fatty acids, decreases with increasing age. Compositions of eicosapentaenoic acid (EPA) or docosahexaenoic acid (DHA) were found to be positively correlated with serum total cholesterol (T-CHO), compositions of stearic acid (StA) or oleic acid (OlA) were positively correlated with serum triglycerides (TGs), and compositions of linoleic acid (LiA) or arachidonic acid (AA) were negatively correlated with TGs [1].

Further, a comparison of lipid fatty acids based on concentration basis vs composition (weight %) was reported for patients with and without coronary artery disease (CAD), concluding that serum concentrations of lipid fatty acids are more accurate reflections of changes in lipid fatty acids rather than compositions [6]. Concentrations of palmitic acid (PA), StA, OlA, LiA, and AA were found to be higher, and concentrations of EPA were lower in subjects with CAD than in subjects without CAD, whereas compositions of PA and StA were higher and those of EPA and DHA were lower in subjects with CAD [6]. A previous study also reported that people with higher serum levels of saturated fatty acids, n-9 MUFA, and n-6 PUFAs more frequently experienced lacunar or atherosclerotic stroke during middle age (50 to 74 years old) [7]. Serum FA concentrations and compositions might thus be associated with acute ischaemic stroke (AIS). However, limited information is available regarding such parameters and their association with AIS in elderly patients. The aim of our retrospective study was to investigate s-FA concentrations, s-FA compositions, and their correlation with age, serum lipids, and ischaemic stroke subtypes in AIS elderly patients.

## Methods

We conducted a cross-sectional study of acute ischaemic stroke patients aged 50 years or older who 1) were admitted to our institution between September 2015 and March 2017 within 24 h of first stroke onset and then 2) underwent blood evaluations of s-LPs and s-FAs at arrival. We excluded patients who used statins, supplemental n-3 PUFA, fibrates, or ezetimibe at the onset. Additionally, we excluded those with a pre-hospital modified Rankin scale (mRS) score of 3 or more or a body mass index (BMI) less than 18.5, which was defined as severe disability or underweight according to the World Health Organisation (WHO) guidelines, as we determined that possible malnutrition was not appropriate for our investigation.

### Measurements

Serum T-CHO, TG, and HDL-cholesterol (HDL-C) were measured through enzymatic analysis using reagents manufactured by Denka Seiken. A BioMajesty 6050 (JEOL Ltd., Tokyo, Japan) was used for measurements. For this purpose, 1.0 ml of serum was used to analyse fatty acids. Serum fatty acids were analysed at the Safety studies section at BML, Inc. (Tokyo, Japan). Fatty acids were extracted according to the general technique of Bligh and Dyer using tricosanoic acid (Nu-Chek Prep, Inc., MN, USA) as an internal standard. The lipid extracts were hydrolysed and extracted with chloroform and evaporated to dryness under nitrogen. After a 30% potassium methoxide methanol solution (FUJIFILM Wako Pure Chemical Corporation, Osaka, Japan) was added to the residual sample, it was incubated at 100 °C for 5 min and cooled. Boron trifluoride methanol reagent was added and incubated at 100 °C for 10 min for methyl esters. The samples were extracted with hexane before analysis. These samples were analysed with a capillary GC: column (BPX70, 30 m × 0.22 mm ID, 0.25 μm film thickness; SHIMADZU GLC Ltd., Tokyo, Japan). The gas chromatograph was a GC-2010Plus (SHIMADSU Corporation, Kyoto, Japan) equipped with a flame ionisation detector. Operating conditions were as follows: the oven temperature was 50 °C for 0.5 min, and then was raised to 260 °C over 25 min and held for 5 min. Injector temperature was 240 °C, the detector temperature was 280 °C, and helium as the carrier gas was delivered at 1.09 ml/min. The identification of components was based on a comparison of retention times with those of standards (Sigma-Aldrich Japan, Inc., Tokyo, Japan; Nu-Chek Prep, Inc., MN, USA). The concentration of each sample was calculated from the internal standard ratio of the standard.

### Variables

The following fatty acids were examined: SFAs [lauric acid (LaA; C12:0), myristic acid (MyA; C14:0), palmitic acid (PA; C16:0), stearic acid (StA; C18:0)], an n-9 MUFA [oleic acid (OlA; C18:1)], n-6 PUFAs [linoleic acid (LiA; C18:2), di-homo-gamma-linolenic acid (DGLA; C20:3), and arachidonic acid (AA; C20:4)] and n-3 PUFAs [alpha-linolenic acid (AlA; C18:3), eicosapentaenoic acid (EPA; C20:5), and docosahexaenoic acid (DHA; C22:6)]. Low-density lipoprotein (LDL)-cholesterol (LDL-C) was calculated using the Friedewald formula as follows: LDL-C = T-CHO − HDL-C − TG/5. Mean blood pressure (MBP) was calculated using the following formula: DBP + (SBP − DBP) / 3.

### Evaluation

We evaluated concentrations of s-LPs, concentrations of s-FAs and compositions created against total fatty acids of s-FAs (in weight percent; %), and correlation coefficients of s-FA concentrations and compositions with age and s-LPs. We divided age into three groups, specifically 50–64 years as working-age after menopause in females, 65–79 years as probable life expectancy after retirement, and 80 years or older as the oldest age. We also compared concentrations and compositions (%) of s-FAs between age groups and between AIS subtypes of large-artery atherosclerosis (LAA), small-vessel occlusion (SVO; lacunar stroke), cardioembolic (CE) stroke, TIA, and patients for whom AIS subtype was not determined [8].

### Statistical analysis

Normally distributed continuous variables are expressed as means ± standard deviations (SDs) and an unpaired Student’s t-test or analysis of variance (ANOVA) was used to compare unpaired groups. Non-normally distributed continuous variables were expressed as medians and interquartile ranges (IQRs), and a Wilcoxon rank-sum test was used to compare unpaired groups. A multiple comparison test was used to compare all possible pairs among three groups. The Pearson correlation coefficient (r) was used to measure the strength of the linear relationship between normally distributed variables, and the Spearman rank correlation coefficient (r_s_) was used to measure the strength of the relationship between non-normally distributed variables. We defined 0 ≤ |r| < 0.3 or 0 ≤ |r_s_| < 0.3 as no correlation, 0.3 ≤ |r| < 0.5 or 0.3 ≤ |r_s_| < 0.5 as a weak correlation, 0.5 ≤ |r| < 0.7 or 0.5 ≤ |r_s_| < 0.7 as a moderate correlation, and 0.7 ≤ |r| < 0.9 or 0.7 ≤ |r_s_| < 0.9 as a strong correlation. Logistic regression was used to estimate odds ratios (OR) and 95% confidence intervals (CIs) for ischaemic stroke in LAA or SVO groups. A probability (p) value less than 0.05 was considered statistically significant. We used the JMP software program (version 15.0; SAS Institute, Cary, NC, USA) to perform statistical analyses.

## Results

A total of 463 patients with ischaemic stroke were admitted to our stroke centre during the study period. Among them, 129 patients were excluded from our analysis because of a pre-hospital mRS scores of 3 or more, 37 were excluded with a BMI less than 18.5, 57 were excluded because they did not undergo s-FA analysis at admission, and 49 were excluded for using medication for dyslipidaemia. Finally, 191 patients met our inclusion criteria (Table 1). All patients were of East Asian ethnicity and were most likely of Japanese ancestry. Regarding stroke subtype, 52 (27.2%) patients experienced LAA, 50 patients experienced SVO (lacunar stroke), 50 patients (26.2%) experienced CE stroke, five patients (2.6%) experienced TIA, and for 34 (17.8%) patients, the subtype was not determined. BMI was lower in females, concentrations of T-CHO, LDL-C, and HDL-C were higher in females, and levels of DGLA, AA, and DHA were also higher in females (Table 2).

**Table 1:** All patients’ characteristics

	*n* = 191
Age (m ± SD) years	74.4 ± 10.4 (min: 50, max: 104)
Male gender	121 (63.3%)
Height (m ± SD) cm	160.8 ± 9.4
Body Weight (MD, IQR) kg	60.0, 52.5–69.0
BMI (MD, IQR) kg/m2	23.1, 20.7–25.1
MBP (MD, IQR) mmHg	115, 101–128
**Ischemic Stroke**	
**Large-artery Atherosclelosis and Small-vessel Occlusion**	***n*** **= 102**
Large-artery Atherosclelosis (LAA)	*n* = 52
Small-vessel Occlusion (SVO)	*n* = 50
**Cardioembolism and Others**	***n*** **= 89**
Cardioembolism	n = 50
TIA	n = 5
not determined	*n* = 34
**Glucose and Lipids**	
BS (MD, IQR) mg/dl	120, 106–149
HbA1c (MD, IQR) %	5.8, 5.6–6.3
T-CHO (m ± SD) mg/dl	203.4 ± 40.2
LDL-C (m ± SD) mg/dl	121.2 ± 33.5
HDL-C (MD, IQR) mg/dl	56.1, 45.2–69.6
TG (MD, IQR) mg/dl	94.5, 68.8–134.5
**Saturated Fatty Acids**	
lauric acid (LaA) (C12:0) (MD, IQR) μg/ml	1.2, 0.7–2.3
myristic acid (MyA) (C14:0) (MD, IQR) μg/ml	18.7, 13.7–27.2
palmitic acid (PA) (C16:0) (MD, IQR) μg/ml	680.7, 593.6–783.1
stearic acid StA (C18:0) (MD, IQR) μg/ml	203.3, 175.4–233.3
LaA (C12:0) (MD, IQR) %	0.04, 0.03–0.07
MyA (C14:0) (MD, IQR) %	0.64, 0.51–0.88
PA (C16:0) (m ± SD) %	23.7 ± 1.5
StA (C18:0) (m ± SD) %	7.0 ± 0.8
**n-9 Monounsaturated Fatty Acid**	
oleic acid (OlA) (C18:1) (MD, IQR) μg/ml	602.5, 515.8–717.4
OlA (C18:1) (m ± SD) %	21.3 ± 2.5
**n-6 Polyunsaturated Fatty Acids**	
linoleic acid (LiA) (C18:2) (MD, IQR) μg/ml	795.2, 658.2–928.3
dihomogammalinolenic acid (DGLA) (C20:3) (MD, IQR) μg/ml	28.1, 21.6–36.4
arachidonic acid (AA) (C20:4) (MD, IQR) μg/ml	146.1, 122.1–169.9
LiA (C18:2) (m ± SD) %	27.1 ± 3.4
DGLA (C20:3) (m ± SD) %	0.99 ± 0.27
AA (C20:4) (m ± SD) %	5.1 ± 1.1
**n-3 Polyunsaturated Fatty Acids**	
Alpha-linolenic acid (AlA) (C18:3) (MD, IQR) μg/ml	21.3, 15.9–29.3
eicosapentaenoic acid (EPA) (C20:5) (MD, IQR) μg/ml	62.2, 45.7–92.5
docosahexaenoic acid (DHA) (C22:6) (m ± SD) μg/ml	136.9 ± 43.2
AlA (C18:3) (MD, IQR) %	0.72, 0.6–0.96
EPA (C20:5) (MD, IQR) %	2.2, 1.5–3.23
DHA (C22:6) (m ± SD) %	4.7 ± 1.4
EPA / AA ratio (MD, IQR)	0.45, 0.32–0.6
n-6 / n-3 ratio (MD, IQR)	4.25, 3.38–5.2

*BMI* Body mass index, *BS* Blood sugar, *T-CHO* Total cholesterol, *TG* Triglyceride

*LDL-C* Low density lipoprotein cholesterol, *HDL-C* High density lipoprotein cholesterol

*m* Mean, *SD* Standard deviation, *MD* Median, *IQR* Interquartile range

*min* Minimum, *max* Maximum, *MBP* Mean blood pressure

%: weight percent of fatty acids

n-6: n-6 polyunsaturated fatty acids

n-3: n-3 polyunsaturated fatty acids

**Table 2:** Comparison between gender and ischemic stroke subtypes

	Male	Female	*p*	LAA_SVO	CE_O	*p*
	*n* = 121	*n* = 70		*n* = 102	*n* = 89	
Age (m ± SD) years	74.4 ± 10.4	74.4 ± 10.7	ns	71.86 ± 10.46	**77.37 ± 9.68**	0.0002
BMI (MD, IQR) kg/m2	**23.7, 21.6–25.2**	22.3, 20.1–24.8	< 0.05	22.9, 20.7–24.8	23.5, 20.9–25.2	ns
Gender						
Male				62	59	ns
Female				40	30	
**Lipids**						
T-CHO (m ± SD) mg/dl	194.6 ± 39.4	**218.4 ± 37.3**	< 0.0001	**213.9 ± 41.7**	191.2 ± 34.8	< 0.0001
LDL-C (m ± SD) mg/dl	113.5 ± 32.3	**135.6 ± 31.1**	< 0.0001	**128.4 ± 34.8**	113.9 ± 30.4	0.0028
HDL-C (MD, IQR) mg/dl	53.5, 42.9–63.6	**61.8, 50.9–73.9**	0.0023	58.5, 46.4–70.6	55.2, 43.7–67.9	ns
TG (MD, IQR) mg/dl	101, 68–163	87, 70.5–116.3	ns	**106.5, 73–166.8**	85.5, 64–115.8	0.0054
**Saturated Fatty Acids**						
LaA (C12:0) (MD, IQR) μg/ml	1,2, 0.7–2.7	1,2, 0.8–2.0	ns	1.3, 0.7–2.6	1.1, 0.7–1.95	ns
MyA (C14:0) (MD, IQR) μg/ml	17.6, 13.3–29.7	19.2, 14.7–25.3	ns	19.75, 14.48–31.9	17.5, 13.1–23.6	ns
PA (C16:0) (MD, IQR) μg/ml	678.6, 592.8–829.4	702.1, 595.4–756.9	ns	**717.15, 605.0–833.1**	648.4, 577.2–724.3	0.0023
StA (C18:0) (MD, IQR) μg/ml	195.9, 167.4–229.6	211.1, 187.9–236.9	ns	**210.4, 183.4–240.4**	193.5, 164.7–221.4	0.003
LaA (C12:0) (MD, IQR) %	0.04, 0.03–0.08	0.04, 0.03–0.07	ns	0.04, 0.03–0.08	0.04, 0.025–0.07	ns
MyA (C14:0) (MD, IQR) %	0.64, 0.495–0.935	0.64, 0.568–0.768	ns	0.65, 0.51–0.933	0.64, 0.51–0.84	ns
PA (C16:0) (m ± SD) %	**24.05 ± 1.44**	23.13 ± 1.39	< 0.0001	23.7 ± 1.6	23.7 ± 1.3	ns
StA (C18:0) (m ± SD) %	6.89 ± 0.77	7.11 ± 0.75	ns	6.95 ± 0.79	6.99 ± 0.08	ns
**n-9 Monounsaturated Fatty Acid**						
OlA(C18:1) (MD, IQR) μg/ml	615.4, 513.6–734.6	583.0, 514.8–712.9	ns	**638.2, 527.8–754.5**	567.5, 499.4–649.3	0.0086
OlA (C18:1) (m ± SD) %	**21.63 ± 2.52**	20.59 ± 2.30	0.005	21.29 ± 2.46	21.21 ± 2.54	ns
**n-6 Polyunsaturated Fatty Acids**						
LiA (C18:2) (MD, IQR) μg/ml	782.3, 643.7–919	822.4, 681.0–940.4	ns	**844.65, 698.7–977.48**	728.5, 636.45–883.7	0.003
DGLA (C20:3) (MD, IQR) μg/ml	26.9, 21.0–34.8	**31.5, 23.6–38.2**	0.018	**32, 24.3–38.33**	24.4, 18.9–31.15	< 0.0001
AA (C20:4) (MD, IQR) μg/ml	142.2, 114.9–162.3	**156.3, 136.1–175.1**	0.0034	**155.7, 132.53–182.18**	136.3, 114.05–158.15	< 0.0001
LiA (C18:2) (m ± SD) %	27.036 ± 3.4	27.31 ± 3.35	ns	27.06 ± 3.46	27.22 ± 3.30	ns
DGLA (C20:3) (m ± SD) %	0.95 ± 0.25	**1.05 ± 0.29**	0.0125	**1.04 ± 0.28**	0.93 ± 0.24	0.0028
AA (C20:4) (m ± SD) %	4.94 ± 1.17	**5.27 ± 1.00**	0.0452	5.18 ± 1,15	4.92 ± 1.07	ns
**n-3 Polyunsaturated Fatty Acids**						
AlA (C18:3) (MD, IQR) μg/ml	21.5, 15.3–30.9	20.9, 16.4–27.5	ns	21.7, 16.13–31.13	20.8, 14.75–27.55	ns
EPA(C20:5) (MD, IQR) μg/ml	59.3, 44.7–90.1	73.2, 48.6–94.1	ns	63, 45.68–92.88	62.5, 45.25–92.85	ns
DHA (C22:6) (m ± SD) μg/ml	130.1 ± 43.5	**148.6 ± 40.4**	0.0012	139.3 + 4.3	134.15 + 4.58	ns
AlA (C18:3) (MD, IQR) %	0.7, 0.6–0.99	0.72, 0.59–0.89	ns	0.71, 0.59–0.99	0.72, 0.61–0.95	ns
EPA (C20:5) (MD, IQR) %	2.1, 1.43–3.15	2.29, 1.67–3.44	ns	2.05, 1.41–3.15	2.4, 1.69–3.29	ns
DHA (C22:6) (m ± SD) %	4.47 ± 1.32	**5.00 ± 1.38**	0.0094	4.53 ± 1.41	4.81 ± 1.30	ns
EPA / AA ratio (MD, IQR)	0.45, 0.315–0.6	0.455, 0.343–0.6	ns	0.43, 0.28–0.59	0.48, 0.365–0.68	ns
n-6 / n-3 ratio (MD, IQR)	4.31, 3.445–5.295	4.1, 3.14–5.228	ns	4.355, 3.428–5.653	4.16, 3.21–4.99	ns

*m* Mean, *SD* Standard deviation, *MD* Median, *IQR* Interquartile range, *%* Weight percent of fatty acids, *p* Probability, *BMI* Body Mass Index, *T-CHO* Total cholesterol, *TG* Triglyceride, *LDL-C* Low density lipoprotein cholesterol, *HDL-C* High density lipoprotein cholesterol, *LAA_SVO* Large-artery atherosclerosis and small-vessel occlusion, *CE_O* Cardioembolism and others, *LaA* Lauric acid, *MyA* Myristic acid, *PA* Palmitic acid, *StA* Stearic acid, *OlA* Oleic acid, *LiA* Linoleic acid, *DGLA* Dihomogammalinolenic acid, *AA* Arachidonic acid, *ALA* Alpha-linolenic acid, *EPA* Eicosapentaenoic acid, *DHA* Docosahexaenoic acid, *n-6* n-6 polyunsaturated fatty acids, *n-3* n-3 polyunsaturated fatty acids

Patients with LAA or SVO (LAA_SVO) ischaemic stroke subtypes due to arteriosclerosis were younger and had higher concentrations of T-CHO, LDL-C, TG, PA, StA, OlA, LiA, DGLA, and AA than patients with an ischaemic stroke subtype of CE or others (CE_O). However, there were no differences in BMI, sex, AlA, EPA, and DHA concentrations and no differences in s-FAs compositions, except for that of DGLA, between LAA_SVO and CE_O groups (Table 2).

Serum T-CHO and TG were weakly negatively correlated with age (Table 3). Serum concentrations of PA, StA, OlA, LiA, DGLA, AA, and AlA were weakly negatively correlated with age and compositions of SFAs, LiA, AA, and AlA were not correlated with age. Compositions of OlA and DGLA and the EPA/AA ratio were weakly negatively correlated with age. Compositions of EPA and DHA and the n-6/n-3 ratio were weakly positively correlated with age. Most s-FA concentrations were weakly negatively correlated with age, and most s-FA compositions had no correlations with age in our patients (Table 3). Serum concentrations of StA, LiA, and AA showed positive correlations with T-CHO, concentrations of SFAs, LiA, and AlA were positively correlated with TG, and the concentration of OlA was strongly correlated with TG (r_s_ = 0.7661). In contrast, no serum FA concentrations exhibited correlations with LDL-C or HDL-C (Table 3). Further, compositions of MyA and OlA showed positive correlations with TG, whereas no serum FA compositions were correlated with LDL-C or HDL-C (Table 3).

**Table 3:** Correlation coefficient between serum fatty acids, serum lipids concentration and age

	T-CHO		TG	*p*	LDL-C		HDL-C		Age		BMI	*p*
	CC	p	CC		CC	p	CC	p	CC	p	CC	
**Age**	−0.1998P	0.0057	− 0.1921	0.0079	− 0.0322P	ns	−0.1229	ns				
**BMI**	−0.0914	ns	0.1917	< 0.01	−0.0668	ns	−0.2742	< 0.001	−0.2139	< 0.01		
**Saturated Fatty Acids**												
**Concentration (μg/ml)**												
LaA (C12:0)	0.2622	0.0003	**0.5601**	< 0.0001	0.1327	ns	−0.0102	ns	− 0.1301	ns	0.0677	ns
MyA (C14:0)	0.2896	< 0.0001	**0.6532**	< 0.0001	0.1107	ns	−0.002	ns	− 0.1290	ns	0.0828	ns
PA (C16:0) (MD, IQR)	0.4785	< 0.0001	**0.6864**	< 0.0001	0.1973	0.0064	0.1404	ns	− 0.2543	4E-04	0.1255	ns
StA (C18:0)	**0.5655**	< 0.0001	0.4816	< 0.0001	0.2884	< 0.0001	0.3454	< 0.0001	−0.2854	< 0.0001	0.0545	ns
(C12:0) + (C14:0) + (C16:0) + (C18:0)	**0.5081**	< 0.0001	**0.6705**	< 0.0001	0.218	0.0025	0.1927	0.0077	−0.2646	2E-04	0.1136	ns
**Composition (%)**												
LaA (C12:0)	0.1222	ns	0.4638	< 0.0001	0.0369	ns	−0.0648	ns	− 0.0757	ns	0.0689	ns
MyA (C14:0)	0.0872	ns	**0.5203**	< 0.0001	−0..0141	ns	−0.1013	ns	−0.187	ns	0.0593	ns
PA (C16:0)	−0.0922P	ns	0.3202	< 0.0001	−0.1631P	0.0245	−0.1917	0.008	−0.0127P	ns	0.1436	< 0.05
StA (C18:0)	0.1228P	ns	−0.2581	0.0003	−0.153P	0.0351	0.3434	< 0.0001	−0.0012P	ns	−0.0569	ns
**n-9 Monounsaturated Fatty Acid**												
**Concentration (μg/ml)**												
OlA (C18:1)	0.3245	< 0.0001	**0.7661**	< 0.0001	0.092	ns	−0.0331	ns	− 0.3161	< 0.0001	0.1873	< 0.01
**Composition (%)**												
OlA (C18:1)	−0.1742P	0.0162	**0.5635**	< 0.0001	−0.2482P	0.0006	−0.4201	< 0.0001	−0.2296P	0.001	0.2003	< 0.01
**n-6 Polyunsaturated Fatty Acids**												
**Concentration (μg/ml)**												
LiA (C18:2)	**0.5459**	< 0.0001	**0.5088**	< 0.0001	0.3348	< 0.0001	0.2103	0.0036	−0.3247	< 0.0001	0.0708	ns
DGLA (C20:3)	0.4914	< 0.0001	0.3716	< 0.0001	0.3294	< 0.0001	0.1892	0.0089	−0.3069	< 0.0001	0.0579	ns
AA (C20:4)	**0.6393**	< 0.0001	0.1561	0.0315	0.4343	< 0.0001	0.4003	< 0.0001	− 0.2295	0.001	− 0.0400	ns
(C18:2) + (C20:3) + (C20:4)	**0.6259**	< 0.0001	0.4824	< 0.0001	0.3859	< 0.0001	0.2789	< 0.0001	−0.3422	< 0.0001	0.0540	ns
**Composition (%)**												
LiA (C18:2)	0.1421P	ns	−0.1488	0.0405	0.1592p	0.0282	0.1023	ns	0.0933p	ns	−0.0329	ns
DGLA (C20:3)	0.1874P	0.0096	−0.0154	ns	0.1779P	0.014	0.1323	ns	−0.1514P	0.037	0.0066	ns
AA (C20:4)	0.1300P	ns	−0.4858	< 0.0001	0.1475P	0.0423	0.2975	< 0.0001	0.0589P	ns	−0.1795	< 0.05
**n-3 Polyunsaturated Fatty Acids**												
**Concentration (μg/ml)**												
AlA (C18:3)	0.2221	0.0021	**0.6345**	< 0.0001	0.0813	ns	−0.039	ns	−0.2146	0.003	0.0750	ns
EPA (C20:5)	0.155	0.0327	−0.0043	ns	0.1439	0.0476	0.1443	0.0471	0.1094	ns	−0.0071	ns
DHA (C22:6)	0.2727P	0.0001	0.1844	0.0109	0.1973P	0.0064	0.1329	ns	0.1089P	ns	−0.0822	ns
(C18:3) + (C20:5) + (C22:6)	0.2516	0.0005	0.1919	0.008	0.1735	0.0167	0.1331	ns	0.0571	ns	−0.0296	ns
**Composition (%)**												
AlA (C18:3)	0.0016	ns	0.4610	< 0.0001	−0.0506	ns	−0.1434	0.0484	−0.1117	ns	0.0157	ns
EPA (C20:5)	−0.0639	ns	−0.2598	0.0003	0.0219	ns	0.0629	ns	0.2350	0.001	−0.0248	ns
DHA (C22:6)	−0.1204P	ns	−0.2505	0.0005	0.0234P	ns	0.0084	ns	0.3400P	< 0.0001	−0.1455	< 0.05
**EPA / AA ratio**	−0.1367	ns	−0.0847	ns	−0.0554	ns	−0.0419	ns	0.2094	0.004	0.0082	ns
**n-6 / n-3 ratio**	0.1498	0.0392	0.1226	ns	0.0773	ns	0.0251	ns	−0.2497	5E-04	0.0456	ns

*m* Mean, *SD* Standard deviation, *MD* Median, *IQR* Interquartile range, *%* Weight percent of fatty acids, *p* Probability, *BMI* Body Mass Index, *T-CHO* Total cholesterol, *TG* Triglyceride, *LDL-C* Low density lipoprotein cholesterol, *HDL-C* High density lipoprotein cholesterol, *CC* Spearman’s rank correlation coefficient except superscript “P”, Superscript “P” meaning Pearson’s correlation coefficient, *LaA* Lauric acid, *MyA* Myristic acid, *PA* Palmitic acid, *StA* Stearic acid, *OlA* Oleic acid, *LiA* Linoleic acid, *DGLA* Dihomogammalinolenic acid, *AA* Arachidonic acid, *ALA* Alpha-linolenic acid, *EPA* Eicosapentaenoic acid, *DHA* Docosahexaenoic acid, *n-6* n-6 polyunsaturated fatty acids, *n-3* n-3 polyunsaturated fatty acids

Patients aged 50–64 years had the highest concentrations of TG, PA, StA, OlA, LiA, DGLA, and AA among the three age groups, and patients aged 80 years or older had the highest compositions of EPA (2.47%) and DHA (5.14%) (Table 4). Twenty-five (78.1%) of 32 patients aged 50–64 years suffered from LAA_SVO and these 25 patients had higher concentrations of TG (MD, IQR: 173, 104–223 mg/dl), PA (777.8, 704.5–960.5 μg/ml), StA (229.4, 208.6–280.05 μg/ml), OlA (742.5, 637.45–865.85 μg/ml), LiA (896.8, 806.4–1007.75 μg/ml), DGLA (36.8, 28.9–48.35 μg/ml), and AA (172.4, 146.4–204.5 μg/ml) than the seven patients with CE_O; however, there were no differences in the compositions of EPA and DHA or the EPA/AA and n-6/n-3 ratios between LAA_SVO and CE_O groups among the 68 patients aged 80 or older (Table 5). These oldest 68 patients had the highest compositions of EPA and DHA; however, there were no differences in such compositions between LAA_SVO and CE_O groups (Table 5). With adjustments for age, TCHO, and TG by logistic regression analysis, AA (OR = 1.011, 95% CI, 0.999–1.012), DGLA (OR = 1.042, 95% CI, 0.998–1.089), and T-CHO (OR = 1.011, 95% CI, 0.999–1.024) were weakly associated with LAA_SVO, rather than CEO_O, although TG (OR = 1.013, 95% CI, 1.003–1.024) and age (OR = 0.961, 95% CI, 0.927–0.993) were associated with LAA_SVO, rather than CEO_O.

**Table 4:** Comparison of lipids and fatty acids with significant correlation with age among age groups

	50 = < Age < 65	65 = < Age < 80	80 = < Age	
	*n* = 32	*n* = 91	*n* = 68	*p*
Age (m ± SD) years	57.8 ± 4.1	72.3 ± 4.3	85.2 ± 4.5	
**Lipids**				
T-CHO (m ± SD) mg/dl	213.9 ± 42.9	203.7 ± 41.2	197.9 ± 36.9	ns
TG (MD, IQR) mg/dl	**145.5, 65.5–217.8 ab**	92, 69–134 a	92, 69–116 b	0.0118
**Saturated Fatty Acids**				
LaA (C12:0) (MD, IQR) μg/ml	1.65, 0.8–3.65	1,2, 0.7–2.4	1.1, 0.7–1.78	ns
MyA (C14:0) (MD, IQR) μg/ml	21.9, 14.7–38.1	17.6, 13.8–27.2	17.5, 12.9–23.9	ns
PA (C16:0) (MD, IQR) μg/ml	**763.9, 666.9–960.5 ac**	678.8, 580.7–828.7 a	647.7, 577.1–735.6 c	0.001
StA (C18:0) (MD, IQR) μg/ml	**222.1, 196.6–258.8 bc**	207.4, 175.1–241.1 b	185, 165.9–215.3 c	0.001
**n-9 Monounsaturated Fatty Acid**				
OlA (C18:1) (MD, IQR) μg/ml	**718.2, 614.9–863.3 bd**	**604.8, 515.8–723.6 ab**	550.3, 494.0–644.6 ad	< 0.0001
OlA (C18:1) (m ± SD) %	**22.3 ± 2.4 b**	21.3 ± 2.4 a	20.7 ± 2.5 ab	0.0046
**n-6 Polyunsaturated Fatty Acids**				
LiA (C18:2) (MD, IQR) μg/ml	**882.4, 783.3–1003.8 d**	**826.2, 692.9–957.4 c**	697.4, 636.2–845.5 cd	< 0.0001
DGLA (C20:3) (MD, IQR) μg/ml	**35.6, 26.9–46.7 ac**	28.7, 21.6–36.5 a,a2	25.3, 19.7–31.2 a2,c	0.0006
AA (C20:4) (MD, IQR) μg/ml	**156.6, 144.1–198.2 ab**	144.7, 122.1–169.9 a	140.6, 120–162.8 b	0.0168
DGLA (C20:3) (m ± SD) %	1.1 ± 0.5	0.99 ± 0.29	0.96 ± 0.26	ns
**n-3 Polyunsaturated Fatty Acids**				
AlA (C18:3) (MD, IQR) μg/ml	**23.1, 18.0–31.2 b**	**23, 16.3–32.6 b2**	18.4, 13.9–23.5 b,b2	0.0026
EPA (C20:5) (MD, IQR) %	1.535, 0.843–2.71 ab	**2.11, 1.54–3.15 a**	**2.47, 1.77–3.54 b**	0.0048
DHA (C22:6) (m ± SD) %	3.88 ± 1.34 bd	**4.58 ± 1.36 b,b2**	**5.14 ± 1.18 b2,d**	< 0.0001
EPA / AA ratio (MD, IQR)	0.325, 0.185–0.525 ab	**0.46, 0.34–0.6 a**	**0.48, 0.353–0.718 b**	0.0115
n-6 / n-3 ratio (MD, IQR)	**4.84, 3.693–7.598 b**	**4.36, 3.42–5.46 a**	3.765, 3.00–4.595 ab	0.0036

*m* Mean, *SD* Standard deviation, *MD* Median, *IQR* Interquartile range, *%* Weight percent of fatty acids, *T-CHO* Total cholesterol, *TG* Triglyceride, *p* Probability, a,a2: < 0.05, b,b2: < 0.01, c: < 0.001, d:< 0.0001, *LaA* Lauric acid, *MyA* Myristic acid, *PA* Palmitic acid, *StA* Stearic acid, *OlA* Oleic acid, *LiA* Linoleic acid, *DGLA* Dihomogammalinolenic acid, *AA* Arachidonic acid, *ALA* Alpha-linolenic acid, *EPA* Eicosapentaenoic acid, *DHA* Docosahexaenoic acid, *EPA* Eicosapentaenoic acid, *AA* Arachidonic acid, *n-6* n-6 polyunsaturated fatty acids, *n-3* n-3 polyunsaturated fatty acids,

**Table 5:** Comparison of lipids and fatty acids with significant correlation between age groups and ischaemic stroke subtypes

	Age Groups								
	50 = < Age < 65 (*n* = 32)		*p*	65 = < Age < 80 (*n* = 91)		*p*	80 = < Age (*n* = 68)		*p*
	LAA_SVO (*n* = 25) 78.1%	CE_O (*n* = 7) 21.9%		LAA_SVO (*n* = 48) 52.7%	CE_O (*n* = 43) 47.3%		LAA_SVO (*n* = 29) 42.6%	CE_O (*n* = 39) 57.4%	
Age (m ± SD) years	58.0 ± 3.9	57.0 ± 5.2	ns	71.5 ± 4.3	73.1 ± 4.2	ns	84.4 ± 4.0	85.7 ± 4.9	ns
**Lipids**									
T-CHO (m ± SD) mg/dl	**224.2 ± 39.9**	177.3 ± 33.7	0.0083	211.3 ± 45.5	195.3 ± 34.3	ns	**209.2 ± 36.2**	189.2 ± 35.5	0.0269
TG (MD, IQR) mg/dl	**173, 104–223**	57, 48–180	0.034	93.5, 72.3–134	90, 67–134	ns	100, 66.5–138.5	86, 70.5–107	ns
**Saturated Fatty Acids**									
LaA (C12:0) (MD, IQR) μg/ml	1.8, 0.95–4.35	1.1, 0.3–1.7	0.068	1,2, 0.73–2.38	1,2, 0.7–2.6	ns	1.0, 0.65–2.2	1.1, 0.8–1.6	ns
MyA (C14:0) (MD, IQR) μg/ml	**23.9, 16.35–39.55**	13, 11.7–22.5	0.0425	18.3, 13.73–27.73	17.6, 14–27.2	ns	18.2, 13.0–30.95	17.5, 12.4–22.1	ns
PA (C16:0) (MD, IQR) μg/ml	**777.8, 704.5–960.5**	661.6, 535.1–718.4	0.024	679.95, 573.48–814.28	674.4, 597–833.1	ns	**728, 586.95–776.7**	621.4, 577–700.3	0.0153
StA (C18:0) (MD, IQR) μg/ml	**229.4, 208.6–280.05**	168.6, 137.3–195.9	0.0051	209.25, 186.7–240.88	201.1, 174.2–243.4	ns	185.6, 176.25–224.9	184.3, 160.9–212.1	ns
**n-9 Monounsaturated Fatty Acid**									
OlA (C18:1) (MD, IQR) μg/ml	**742.5, 637.45–865.85**	601.8, 496.5–640.1	0.0381	602.65, 516.78–716.73	604.8, 506–793.4	ns	593.9, 499.35–692.75	541.1, 491.5–618.6	ns
OlA (C18:1) (m ± SD) %	22.1 ± 2.37	23.2 ± 2.5	ns	21.36 ± 2.58	21.26 ± 2.28	ns	20.51 ± 2.19	20.79 ± 2.68	ns
**n-6 Polyunsaturated Fatty Acids**									
LiA (C18:2) (MD, IQR) μg/ml	**896.8, 806.4–1007.75**	697.5, 586.2–882.2	0.0213	804.35, 687.48–999.63	833.7, 698.4–932.7	ns	**777.3, 666.85–866.1**	664, 619.9–728.5	0.0182
DGLA (C20:3) (MD, IQR) μg/ml	**36.8, 28.9–48.35**	22, 14.9–36.9	0.0448	31.7, 24.23–37.3	28.3, 18.6–36.1	ns	**27.4, 21.45–36**	23.1, 19.6–28	0.0252
AA (C20:4) (MD, IQR) μg/ml	**172.4, 146.4–204.5**	116.4, 106.2–161	0.0189	147.0, 130.8–175.4	144.4, 113.1–158.9	ns	**153, 123.85–172.25**	133.1, 116.2–157.5	0.0401
DGLA (C20:3) (m ± SD) %	1.08 ± 0.23	0.99 ± 0.33	ns	1.04 ± 0.29	0.93 ± 0.27	ns	1.03 ± 0.31	0.91 ± 0.20	ns
**n-3 Polyunsaturated Fatty Acids**									
AlA (C18:3) (MD, IQR) μg/ml	23.3, 19.05–31.4	21.8, 13.7–26.3	ns	21.7, 15.3–32.93	24, 16.4–32.5	ns	19.0, 14.15–25.45	17.3, 13.3–22.4	ns
EPA (C20:5) (MD, IQR) %	1.43, 0.965–2.845	1.93, 0.61–2.74	ns	2.055, 1.50–2.835	2.22, 1.65–3.26	ns	2.23, 1.595–3.845	2.54, 1.92–3.37	ns
DHA (C22:6) (m ± SD) %	3.956 ± 1.35 b	3.60 ± 1.36 ab	ns	4.515 ± 1.458	4.64 ± 1.26	ns	5.05 ± 1.19	5.21 ± 1.18	ns
EPA / AA ratio (MD, IQR)	0.32, 0.19–0.53	0.35, 0.15–0.54	ns	0.455, 0.325–0.578	0.46, 0.37–0.67	ns	0.43, 0.305–0.725	0.48, 0.38–0.7	ns
n-6 / n-3 ratio (MD, IQR)	4.72, 3.625–7.255	5.11, 3.97–7.81	ns	4.49, 3.448–5.625	4.3, 3.38–5.18	ns	4.03, 3.045–4.98	3.73, 2.91–4.55	ns

*m* Mean, *SD* Standard deviation, *MD* Median, *IQR* Interquartile range, *%* Weight percent of fatty acids, *p* Probability, *T-CHO* Total cholesterol, *TG* Triglyceride, *LAA_SVO* Large-artery atherosclerosis and small-vessel occlusion, *CE_O* Cardioembolism and others, *LaA* Lauric acid, *MyA* Myristic acid, *PA* Palmitic acid, *StA* Stearic acid, *OlA* Oleic acid, *LiA* Linoleic acid, *DGLA* Dihomogammalinolenic acid, *AA* Arachidonic acid, *ALA* Alpha-linolenic acid, *EPA* Eicosapentaenoic acid, *DHA* Docosahexaenoic acid, *n-6* n-6 polyunsaturated fatty acids, *n-3* n-3 polyunsaturated fatty acids

## Discussion

Our results of elderly AIS patients demonstrated that concentrations, but not compositions, of PA, OlA, and LiA are significantly correlated with age, TG, and ischaemic stroke subtypes. Patients with LAA_SVO were younger and had higher concentrations of T-CHO, TG, PA, StA, OlA, LiA, DGLA, and AA among patients aged 50 years or older, whereas there were no differences in the compositions of s-FAs, except for that of DGLA, between LAA_SVO and CE_O groups. Although no fatty acid concentrations were significant independent predictors of LAA_SVO, as compared to the predictive power of CE_O, AA and DGLA concentrations had a weak association with LAA_SVO rather than CE_O. Compositions of EPA and DHA were not related to ischaemic stroke subtype but rather were associated with age at onset.

A previous study reported that TG concentration had a stronger relationship with n-6 PUFA than T-CHO concentration in healthy Japanese individuals with normal serum T-CHO and TG levels, and that correlation coefficient between TG and n-6 PUFA was negative, specifically, − 0.417 in men and − 0.330 in women [1]. In our elderly patients, the Spearman rank correlation coefficient comparing TG and LiA was positive, specifically 0.5088 at AIS onset. Correlations between TG and n-6 PUFA were quite different between young, healthy individuals. and elderly patients at AIS onset. A previous study reported that serum palmitic and oleic acid were associated with an increased incidence of incidental atherothrombotic, lacunar, and ischemic strokes of undetermined cause compared to that with matched controls, and that DHA and arachidonic acid were associated with a decreased incidence of ischaemic stroke [9]. However, that was a case-control study in which fasting blood samples were collected from all observational study participants, and their values of serum fatty acids were not reported at AIS onset [9]. Concentrations of PA, StA, and OlA were higher in the LAA_SVO group than in the CE_O group in our elderly AIS patient cohort, although patients with CE_O were not the matched controls but were ischaemic stroke patients.

A previous study reported that higher concentrations of serum n-6 PUFAs and lower levels of MUFAs are strongly associated with lower TG levels [4]. However, in our study, higher concentrations of n-6 PUFAs were associated with higher TG levels. A previous study also reported that mean compositions of PA, OlA, LiA, EPA, and DHA in healthy Japanese male subjects aged 50 to 59 years were 25, 21.3, 27.3, 2.4, and 4.0%, respectively [1], which were almost the same as those in our male patients (Table 2). A previous study reported that the mean concentration of LiA was 2233.8 μmol/L (626.5 μg/ml) in healthy young adults in their 20s [10], which was almost the same as the concentration detected in our patients. In contrast, a previous study reported that in US patients with CAD at an average age of 47 ± 5 years, the mean concentrations of PA, StA, OlA, LiA, DGLA, AA, EPA, and DHA were 823, 13, 1336, 4358, 33, 688, 67, and 40 μg/ml, respectively, and the mean compositions of PA, StA, OlA, LiA, DGLA, AA, EPA, and DHA were 11.3, 0.2, 18.4, 58.6, 0.4, 9.4, 0.9, and 0.5%, respectively [6]. Their concentrations of OlA, LiA, and AA and compositions of LiA and AA were much higher, but compositions of OlA, EPA, and DHA were much lower, than those in our patients (Table 1). Compared to those in our patients, concentrations of 4358 μg/ml of LiA and 688 μg/ml of AA were extremely high, and in that previous study, atherosclerotic CAD typically occurred in patients in their 40s. Our patients with LAA_SVO due to arteriosclerosis were younger among the cohort, and they also had higher concentrations of LiA and AA. Even though the concentration of a fatty acids might be high, its proportion could be low because the proportion is relative. If the concentration of one variable increases, the composition of the other variables has to decrease. Composition, therefore, is not appropriate to identify driving factors. Further, serum concentrations of fatty acids can be more easily interpreted in metabolic and therapeutic terms, as reported previously. Our study was retrospective and cross-sectional, and there was no control group. Therefore, it was not determined whether s-FA concentrations in patients with LAA_SVO were high or whether compositions of EPA and DHA were high or low, compared to those in hypothetical control subjects.

Previous studies have reported that n-3 PUFA might reduce the incidence of CAD or stroke or the mortality associated with cardiovascular disease [11–15]. The administration of highly-purified EPA appeared to reduce the risk of recurrent stroke in a Japanese population of hypercholesterolemic patients receiving low-dose statin therapy [13]. In contrast, SFAs might increase the risk factors associated with these conditions [16]. A fish diet decreases very-low-density lipoprotein (VLDL) but increases both low-density lipoprotein (LDL) and high-density lipoprotein (HDL) in healthy human subjects [3]. Further, dietary SFAs were found to increase serum T-CHO, and it was shown that dietary n-6 PUFAs lower serum T-CHO [17, 18]. LiA-rich food can also lower T-CHO [5]. However, in our patients, the concentration of LiA was strongly positively correlated with T-CHO and TG levels. Dietary fatty acids with lower amounts of PA, StA, OlA, and n-6 PUFAs and higher compositions of EPA and DHA might also reduce the incidence of ischaemic stroke associated with LAA_SVO and in the oldest age group, although the appropriate dietary intake of fatty acids to prevent ischaemic stroke cannot be confirmed based on the results of our study. Serum fatty acids at AIS onset are significantly influenced by recent dietary intake, and it is important to assess what types and quantities of meats and fish were consumed and what kind and volume of vegetable oils were used a few days before AIS onset. The assessment of dietary intake might lead to a recommendation for the appropriate dietary intake of fatty acids to prevent ischaemic stroke. If the appropriate intake of fatty acids is identified, a prospective randomised control study is required to address this.

## Conclusions

In AIS elderly patients, concentrations, rather than compositions, of PA, OlA, and LiA correlated with age, TG, and ischaemic stroke subtype. Further, patients with LAA_SVO were younger and had higher concentrations of PA, OlA, and LiA than those with CE_O, although serum fatty acids were not independent predictors of LAA_SVO, as compared to the predictive power of CE_O. There were no differences in s-FA compositions between LAA_SVO and CE_O groups. Higher compositions of EPA and DHA were associated with the oldest age. Further studies are warranted to determine the appropriate dietary intake of fatty acids for AIS prevention and long life.

## Data Availability

Raw data can be made available upon request to the corresponding author.

## Abbreviations

AA: Arachidonic acid
AIA: Alpha-linolenic acid
BMI: Body mass index
CAD: Coronary artery disease
CE: Cardioembolic stroke
DBP: Diastolic blood pressure
DGLA: Di-homo-gamma-linolenic acid
DHA: Docosahexaenoic acid
EPA: Eicosapentaenoic acid
FA: Fatty acid
HDL-C: High-density lipoprotein cholesterol
LAA: Large-artery atherosclerosis
LaA: Lauric acid
LDL-C: Low-density lipoprotein cholesterol
LiA: Linoleic acid
MBP: Mean blood pressure
mRS: Modified Rankin scale
MyA: Myristic acid
n-3 PUFA: N-3 polyunsaturated fatty acid
n-6 PUFA: N-6 polyunsaturated fatty acid
n-9 MUFA: N-9 monounsaturated fatty acid
OlA: Oleic acid
PA: Palmitic acid
SBP: Systolic blood pressure
SFA: Saturated fatty acid
s-FA: Serum fatty acid
s-LP: Serum lipids
StA: Stearic acids
SVO: Small-vessel occlusion (lacunar stroke)
T-CHO: Total cholesterol
TG: Triglycerides
TIA: Transient ischaemic attack
VLDL: Very-low-density-lipoprotein
